# 3-Mercaptopropionic acid as a potential biocontrol factor contributes to plant protection efficacy of *Pseudomonas protegens* CHA0

**DOI:** 10.3389/fmicb.2026.1726349

**Published:** 2026-02-13

**Authors:** Arisa Kara, Shigemi Seo, Kasumi Takeuchi

**Affiliations:** Institute of Agrobiological Sciences, National Agriculture and Food Research Organization, Tsukuba, Ibaraki, Japan

**Keywords:** antimicrobial metabolites, disease control, pseudomonads, rhizosphere, volatile organic compounds

## Abstract

*Pseudomonas protegens* CHA0 and other fluorescent pseudomonads suppress plant diseases by producing a number of metabolites with antibiotic activities in the rhizosphere. However, limited information is available on the physiological functions of most of these metabolites. We herein describe the detection of 3-mercaptopropionic acid (3-MPA), a thiol compound, based on the metabolome of the supernatant of strain CHA0. 3-MPA exhibited antibiotic activity against oomycete and fungal pathogens and suppressed *Pythium* damping-off and root rot in cucumber. 3-MPA also exhibited antibiotic activity without direct contact with pathogens, indicating the volatilization effect of this compound. We identified a homologous gene encoding 3-MPA dioxygenase in the CHA0 genome and the production level of 3-MPA was elevated in a *3mdo*-negative mutant. Growth inhibitory activity against oomycete and fungal pathogens and plant protection efficacy exhibited by the *3mdo*-negative mutant were stronger than those of wild-type CHA0. These results suggest that 3-MPA is biocontrol factor of strain CHA0.

## 1 Introduction

*Pseudomonas protegens* CHA0 and other root-colonizing pseudomonads are biocontrol bacteria that suppress plant root diseases. Secondary metabolites with antibiotic activity and extracellular enzymes contribute to the efficacy of plant protection by strain CHA0 and other root-colonizing pseudomonads with biocontrol activity. Secondary metabolites, such as 2,4-diacetylphloroglucinol (DAPG), pyoluteorin, pyrrolnitrin, and hydrogen cyanide (HCN), are typical antibiotics produced by this strain (Haas and Keel, 2003). Since the complete genomic sequence of strain Pf-5 has been elucidated, some novel natural products that contribute to disease suppression, such as orfamides, rhizoxin analogs, and 7-hydroxytropolone produced by *P. protegens* and related strains, have been discovered as novel biocontrol factors through genome-mining approaches (Paulsen et al., 2005; Takeuchi et al., 2015; Loper et al., 2016; Muzio et al., 2020). The production of most of these exoproducts is under the control of the Gac/Rsm signal transduction pathway, and the GacS/GacA two-component system initiates this pathway (Haas and Défago, 2005; Kim et al., 2011). When cell population density is high, the GacS sensor kinase is autophosphorylated, enabling the activation of the cognate GacA response regulator. GacA positively regulates the expression of three small RNAs, termed RsmX, RsmY, and RsmZ, which exhibit high affinity for the RNA-binding repressor proteins RsmA and RsmE. These proteins repress the translation of genes required for the synthesis of biocontrol factors; therefore, when small RNAs are induced, they mitigate the translational repression of target genes (Heeb et al., 2002; Valverde et al., 2003; Kay et al., 2005; Reimmann et al., 2005).

A wide range of antibiotic metabolites produced by root-colonizing pseudomonads strengthen bacteria for niche adaptation in the rhizosphere. Volatile organic compounds (VOCs) produced by pseudomonads are more difficult to analyze than soluble, non-volatile antibiotics and exoenzymes; however, their potential for controlling plant diseases has been reported. VOCs with antibiotic activity produced by soil microbes may effectively control soil-borne disease because they spread extensively via air-filled pores in soils (de Boer et al., 2019). Although several pseudomonads have been reported to produce HCN as a potent antifungal VOC, volatiles that have yet to be identified are also involved in disease control (Hunziker et al., 2015; Win et al., 2022). We recently reported that exogenously applied glutamate (Glu) positively regulated the chitinase activity and biocontrol efficacy of strain CHA0 (Takeuchi et al., 2023), suggesting that the production of other antibiotic metabolites also increases in response to Glu. To investigate functional VOCs produced by pseudomonads, we initially examined VOCs in culture solutions of CHA0 incubated with exogenous Glu using a gas chromatography–mass spectrometry (GC/MS)-based non-targeted metabolomics analysis. We identified several compounds, such as fatty acids, as Glu-responsive VOCs. We also detected mercaptopropionic acid (MPA), a thiol compound, in the supernatant of bacterial cultures regardless of whether exogenous Glu was present.

MPA (later identified as 3-MPA) drew our attention because its functions in pseudomonads remain unknown, particularly in plant-microbe and microbe-microbe interactions. To the best of our knowledge, this is the first study to describe the detection of 3-MPA in pseudomonads. Although limited information is available on the bacterial enzymes involved in 3-MPA metabolism, 3-mercaptopropionate dioxygenase (3MDO) has been identified and characterized in *P. aeruginosa* PAO1 (Tchesnokov et al., 2015). 3MDO is a member of the cysteine dioxygenase family and it oxidizes 3-MPA into 3-sulfinopropionic acid. We also investigated the antibiotic activity and function of 3-MPA by utilizing 3-MPA itself and by generating a 3MDO-deficient mutant of *P. protegens* CHA0, which overproduces 3-MPA.

## 2 Materials and methods

### 2.1 Bacterial strains and growth conditions

The bacterial strains and plasmids used are listed in Supplementary Table S1. Strains of *Escherichia coli* and *P. protegens* were routinely grown in nutrient yeast broth [2.5% (wt/vol) nutrient broth and 0.5% (wt/vol) yeast extract] with shaking or on nutrient agar plates [4% (wt/vol) blood agar base and 0.5% (wt/vol) yeast extract] amended with the following antibiotics when required: ampicillin, 100 μg/mL (only for *E. coli*); kanamycin, 50 μg/mL; or tetracycline, 25 μg/mL (100 μg/mL for the selection of *P. protegens*); or chloramphenicol, 10 μg/mL. Inoculation temperatures were 30 °C for *P. protegens* and 37 °C for *E. coli.* In other assays, bacteria were grown in liquid GCM (Maurhofer et al., 1998).

### 2.2 Generation of mutants of *3mdo*, *tst*, and the *gacA* gene

An in-frame deletion of chromosomal *3mdo*, *tst*, and the *gacA* gene of *P. protegens* CHA0 was created as follows. Fragments of *ca.* 700- to 800-bp regions flanking the gene were amplified by PCR with the primer pairs 3mdoUF/3mdoUR and 3mdoDF/3mdoDR for *3mdo*, TstUF/TstUR and TstDF/TstDR for *tst*, or GacAUF/GacAUR and GacADF/GacADR for *gacA*. High-fidelity DNA polymerase KOD plus (Toyobo) and the genomic DNA of *P. protegens* as a template were used for these amplifications. Each of the two corresponding fragments was annealed and amplified as a 1.4- or 1.5-kb fragment using the primer pair 3mdoUF/3mdoDR, TstUF/TstDR, or GacAUF/GacADR. These 1.4- and 1.5-kb fragments were cloned into pCR-BluntII-TOPO (Invitrogen). The inserts obtained were confirmed by sequencing and digested with *Kpn*I and *Hin*dIII, *Bam*HI and *Hin*dIII, and *Eco*RI and *Hin*dIII, respectively. After sequencing, this fragment was subcloned into pME3087 cleaved at the *Kpn*I and *Hin*dIII sites to give pME30873mdo, the *Bam*HI and *Hin*dIII sites to give pME3087tst, and the *Eco*RI and *Hin*dIII sites to give pME3087gacA. These plasmids were mobilized from *E. coli* DH5α to *P. protegens* CHA0 by triparental mating with *E. coli* HB101/pME497. Excision of the vector via a second crossing-over was obtained after the enrichment of tetracycline-sensitive cells, generating the *3mdo*, *tst*, or *gacA* mutant. Oligonucleotides used in the present study are listed in Supplementary Table S2.

### 2.3 Complementation of the *3mdo*-negative mutant

The *3mdo* mutant was complemented with a 1-kb fragment carrying *3mdo*, which had been amplified by PCR with the primers 3mdoCompF and 3mdoCompR (Supplementary Table S2). This fragment was cloned into pCR-BluntII-TOPO (Invitrogen). The inserts obtained were confirmed by sequencing and digested with *Hin*dIII and *Eco*RI. After sequencing, this fragment was subcloned into pME6031 cleaved at *Hin*dIII and *Eco*RI. This plasmid DNA was introduced into the *3mdo* mutant by electroporation.

### 2.4 Metabolomic analysis

*Pseudomonas* *protegens* CHA0 was grown in liquid GCM in Erlenmeyer flasks and incubated at 180 rpm at 30 °C after the inoculation (scaling up from an overnight culture to a fresh culture, 100-fold). Culture solutions were harvested during the idiophase (8 h after the start of the incubation, at OD_600_ of approximately 1.7) and the supernatant (approximately 15 mL) after centrifugation was extracted with 10 mL of ethyl acetate and evaporated to dryness. The remaining residue was dissolved in ethyl acetate and subjected to a GC/MS analysis. Analyses were performed on a GC (7890A, Agilent) coupled with a quadrupole MS (5975C, Agilent) in the scan mode with a range of *m/z* 50 to 500. Separation was performed on a capillary column (HP-1MS, 30-m length, 0.25-mm inner diameter, 0.25-μm thickness; Agilent) with He as the carrier gas at a flow rate of 1 mL/min. The column oven temperature was held at 50 °C for 1 min, increased to 300 °C at 10 °C /min, and held for 5 min. The injection port temperature and transfer line temperature were both 280 °C. Peaks corresponding to volatile compounds were selected and identified by matching their mass spectra with those of reference compounds in a chemical library in the GC–MS apparatus or the mass spectral library of the National Institute of Standards and Technology, United States.

### 2.5 Chemicals

2-MPA, 3-MPA, and monobromobimane (mBr) were purchased from Tokyo Chemical Industry Co. (Tokyo, Japan). 2-MPA and 3-MPA were dissolved in and diluted with water. mBr was dissolved in acetonitrile to a final concentration of 15 mM as a stock solution and stored at −20 °C.

### 2.6 Measurement of MPA

*Pseudomonas* *protegens* CHA0 and its mutants were grown in liquid GCM in Erlenmeyer flasks and incubated at 180 rpm at 30 °C after the inoculation (scaling up from an overnight culture to a fresh culture, 100-fold). The bacterial culture was collected in a microtube 4 or 8 h after the start of the incubation. The mBr derivatization of MPA in bacterial cultures was performed using a previously reported procedure with a slight modification (Allen and White, 2016; Supplementary Figure S1). Ninety microliters of 0.1 moL/L Tris–HCl buffer (pH 9.5) and 25 μL of 1.5 mmoL/L mBr were added to 90 μL of the culture supernatant. Samples were incubated at room temperature for 30 min in the dark. The reaction was stopped by adding 65 μL of 0.2 moL/L sulfosalicylic acid and 230 μL of methanol, and the supernatant after centrifugation was collected and stored at −20 °C until analyzed. The supernatants were diluted with mobile phase buffer (water:acetonitrile:formic acid, 80:20:0.1, vol/vol/vol), and aliquots were subjected to UPLC-MS/MS.

To identify structural isomers of MPA, 90 μL of 100 ng/μL 2-MPA or 3-MPA was derivatized with mBr as described above. Each reaction product was considered to be mBr-labeled MPA with a concentration of 18 ng/μL. mBr-labeled 2-MPA (mBr2MPA) and 3-MPA (mBr3MPA) were mixed and diluted with the mobile phase buffer to concentrations of 0.005, 0.01, 0.05, 0.1, 0.5, and 1 pg/μL. These serially diluted derivatives were also used to prepare standard curves for the assessment of endogenous MPA (Supplementary Figure S2).

Analyses were conducted using a UPLC system (ACUITY H-Class, Waters) coupled to a tandem-quadrupole MS (TQ-S micro, Waters). Separations were performed on a reversed phase UPLC column (Unison UK-C18 HT, 2-mm inner diameter, 100-mm length, 3-μm particle size, Imtakt, Kyoto, Japan) eluting mobile phase buffer at a flow rate of 0.4 mL/min. The column temperature was set to 35 °C.

A mass analysis was performed according to the following conditions: ESI + voltage 3.5 kV; cone gas flow 50 L/h; desolvation gas (N_2_) flow 1,000 L/h; desolvation temperature 350 °C, collision energy 20 eV, and cone voltage 35 V. Full scan spectra were acquired from *m/z* 50 to 500. Quantification was performed in a multiple reaction monitoring mode detecting the transition *m/z* 297 as the parent ion to *m/z* 192 as a product ion. mBr2MPA and mBr3MPA contents were calculated with standard curves using serially diluted concentrations of mBr2MPA and mBr3MPA. Data analyses were performed using the TargetLynx application (Waters). 2-MPA and 3-MPA in samples were expressed as mBr2MPA and mBr3MPA, respectively.

### 2.7 Detection of antibiotic activity

The antibiotic activity of 3-MPA was assessed with *Pythium ultimum* MAFF424594 (reidentified as *Globisporangium oryzicola*; Uzuhashi et al., 2017) or *Fusarium oxysporum* MAFF103054 as the reporter. Oomycete and fungal strains were obtained from the NARO Genebank in Tsukuba. To detect antibiotic activity, strains were grown on potato dextrose agar (PDA) plates (3.9% [wt/vol] potato dextrose agar and 0.3% [wt/vol] agar). A 6-mm mycelial disk of *P. ultimum* or *F. oxysporum* was inoculated on the PDA plate (52 mm in diameter). 3-MPA was diluted to each concentration using sterile water and 30-μL samples were spotted onto a paper disk (8 mm in diameter) at the other end. The distance between *P. ultimum* and the paper disk was set to approximately 17 mm. The plates were incubated at 25 °C in the dark.

To evaluate the antibiotic activity of volatilized 3-MPA, we used the double-plate chamber method (Win et al., 2022). Briefly, 100 μL of 3-MPA solution of each concentration was spotted onto a round piece of filter paper (35 mm in diameter) set in a Petri dish (52 mm in diameter). A 6-mm mycelial disk of *P. ultimum* or *F. oxysporum* was inoculated individually at the center of the PDA plate in the Petri dish (52 mm in diameter). This plate was placed onto the Petri dish with 3-MPA-soaked filter paper such that the two plates faced each other. Plastic tape was used to seal the contact surfaces of the two plates, providing a double-plate chamber, and the plates were incubated at 25 °C in the dark. A double-plate chamber with sterile water instead of 3-MPA was used as the control.

### 2.8 Plant disease suppression assays

The disease suppressive effects of 3-MPA were evaluated as follows. *P. ultimum* MAFF425494 was added as a millet-seed inoculum at 10 g per kg of vermiculite and 1 mL of 200 or 500 mM 3-MPA was added to 300 mL of vermiculite absorbed in 100 mL of water for 4 h in a plastic bag before planting. Control pots received the same amount of sterile water instead of 3-MPA. Three cucumber seedlings (*Cucumis sativus* L. cv. Shin-tokiwajibai) were sown in vermiculite in 125-cm^3^ plastic pots (5 × 5 × 5 cm) containing 50 mL of vermiculite. Seedlings were covered with 15 mL of untreated vermiculite. Cucumber seedlings were incubated in a growth chamber at 60% relative humidity and 26 °C with light for 16 h, followed by an 8-h dark period. After an incubation for 14 days, the disease suppressive effects of 3-MPA were assessed.

To investigate the effects of the *3mdo* mutation, the biocontrol activity of each strain was evaluated as previously described (Takeuchi et al., 2023) with slight modifications. Flasks containing 20 g of vermiculite were planted with three cucumber seedlings each and treated with *P. ultimum* MAFF425494 as a millet-seed inoculum at 10 g per kg of vermiculite. *Pseudomonas strains* were added to vermiculite as a suspension (4 mL per flask) of cells washed once in sterile distilled water to give 2 × 10^7^ CFU per g of vermiculite. Control flasks received the same amount of sterile water. Seedlings were covered with 5 g of untreated vermiculite and flasks were sealed with aerated silicon caps. Cucumber seedlings were incubated in a growth chamber at 60% relative humidity and 26 °C with light for 16 h, followed by an 8-h dark period. No watering was necessary. After an incubation for 7 days, the biocontrol activity of each strain was assessed.

Data in Tables 1, 2, and Supplementary Table S4 represent the means of two individual repetitions of the same experiment, except for the results on fresh weights in Supplementary Table S4, which represent the results of each experiment. Data from both experiments were initially analyzed for a trial-by-treatment interaction by an analysis of variance, which indicated that data from the two independent trials may be pooled, except for the results on fresh weights in Supplementary Table S4. Means were separated using Tukey’s HSD test (at *p* ≤ 0.05). Statistical analyses were performed using R (version 4.2.2).

**Table 1:** The suppression of *Pythium* damping-off and root rot in cucumber by 3-MPA.

3-MPA added*	*Pythium* added*	Per pot		
		Surviving plants (%)**	Shoot fresh weight (g)**	Root fresh weight (g)**
None	−	100 a	0.43 a	0.10 a
None	+	31 c	0.09 c	0.02 c
2 mM	+	67 b	0.17 b	0.04 b
5 mM	+	81 ab	0.19 b	0.05 b

*One milliliter of 3-MPA of 200 or 500 mM was added to 300 mL of vermiculite absorbed in 100 mL of water to make the final concentration of 2 mM or 5 mM 3-MPA in water (50 mL of soil per pot). *Pythium ultimum* was added as the millet-seed inoculum at 10 g/L of vermiculite before planting. Plants were harvested after 14 days. The corresponding photographs of growth features of cucumber seedlings are shown in Supplementary Figure S5. **Data represent the means of two individual repetitions of the same experimental set-up, with 6 replicates (pots contain three cucumber plants) per treatment in each experiment and 12 replicates (pots) in total. Means within the same column followed by different letters (a to c) are significantly different (*p* < 0.05) according to Tukey’s honestly significant difference (HSD) test.

**Table 2:** Effects of the *3mdo* mutant on the suppression of *Pythium* damping-off and root rot of cucumber by *Pseudomonas protegens*.

Bacterial strain added	*Pythium* added*	Per flask		
		Surviving plants (%)**	Shoot fresh weight (g)**	Root fresh weight (g)**
None	−	100 a	0.91 a	0.31 a
CHA0 (wild type)	−	100 a	0.94 a	0.33 a
Δ*3mdo*	−	100 a	0.94 a	0.28 ab
None	+	0 c	0.05 c	0.02 d
CHA0 (wild type)	+	75 b	0.41 b	0.14 c
Δ*3mdo*	+	90 a	0.47 b	0.23 b

*P. protegens CHA0 and the mutant were added at 2 × 10^7^ CFU per gram of vermiculite to 100-mL flasks (20 g of vermiculite per flask) following the planting of three 48-h-old, sterile-grown cucumber seedlings per flask. Prior to planting, *Pythium ultimum* was added, at 10 g per kg of vermiculite, as the millet-seed inoculum. All plants were harvested 7 days later. **Data represent the means of two individual repetitions of the same experimental set-up, with 12 replicates (flasks containing three cucumber plants) per treatment without *P. ultimum* and 16 replicates per treatment with *P. ultimum*. Means within the same column followed by different letters (a to d) are significantly different (*p* < 0.05) according to Tukey’s honestly significant difference (HSD) test.

## 3 Results

### 3.1 Detection of 3-MPA from the culture supernatant of *P. protegens* CHA0

**Figure 1:**
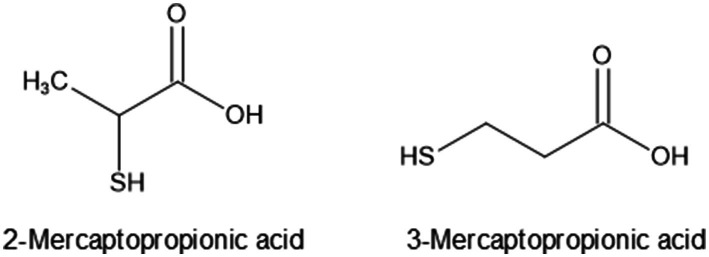
Chemical structures of 2-MPA and 3-MPA.

MPA has two structural isomers, 2-MPA and 3-MPA (Figure 1). To establish which isomer was detected, we attempted to measure endogenous 2-MPA and 3-MPA. Difficulties are associated with directly measuring thiol compounds because of their chemical instability. Monobromobimane (mBr) reacts selectively with thiol groups to form stable sulfur-containing compounds and has been used to quantify low-molecular-weight thiol compounds, such as hydrogen sulfide, glutathione, and MPA, in biological samples (Fahey and Newton, 1987; Winter et al., 2010; Sardi et al., 2013; Salgado et al., 2015; Shen et al., 2015; Allen and White, 2016). Authentic 2-MPA and 3-MPA were incubated with mBr (Supplementary Figure S1), and the reaction products were subjected to ultraperformance liquid chromatography-tandem MS (UPLC-MS/MS). mBr-labeled 2-MPA (mBr2MPA) and 3-MPA (mBr3MPA) were chromatographically separated (Supplementary Figure S3A). A mass fragmentation analysis of these two derivatives confirmed the existence of the parent ion (*m/z* 297) and a product ion (*m/z* 192) derived from the loss of the MPA molecule (Supplementary Figures S3B,C). Therefore, we successfully distinguished between 2-MPA and 3-MPA using the combination of mBr derivatization and UPLC-MS/MS. To assess the endogenous contents of 2-MPA and 3-MPA in strain CHA0, we measured mBr2MPA and mBr3MPA in the spent culture supernatant incubated with mBr. The concentration of mBr2MPA was 56.7 ± 43.3 pg per optical density at 600 nm (OD_600_) while that of mBr3MPA was 1664.4 ± 99.2 pg/OD_600_ after 8 h of cultivation (Supplementary Figure S4; Supplementary Table S3). These results indicate that the majority of MPA analogs produced by strain CHA0 were 3-MPA, supporting previous findings on other bacteria, such as *Methanocaldococcus jannaschii* and Var*iovorax paradoxus* (Bruland et al., 2009; Allen and White, 2016).

### 3.2 3-MPA exhibits antibiotic activities against *Pythium ultimum* and *Fusarium oxysporum*

**Figure 2:**
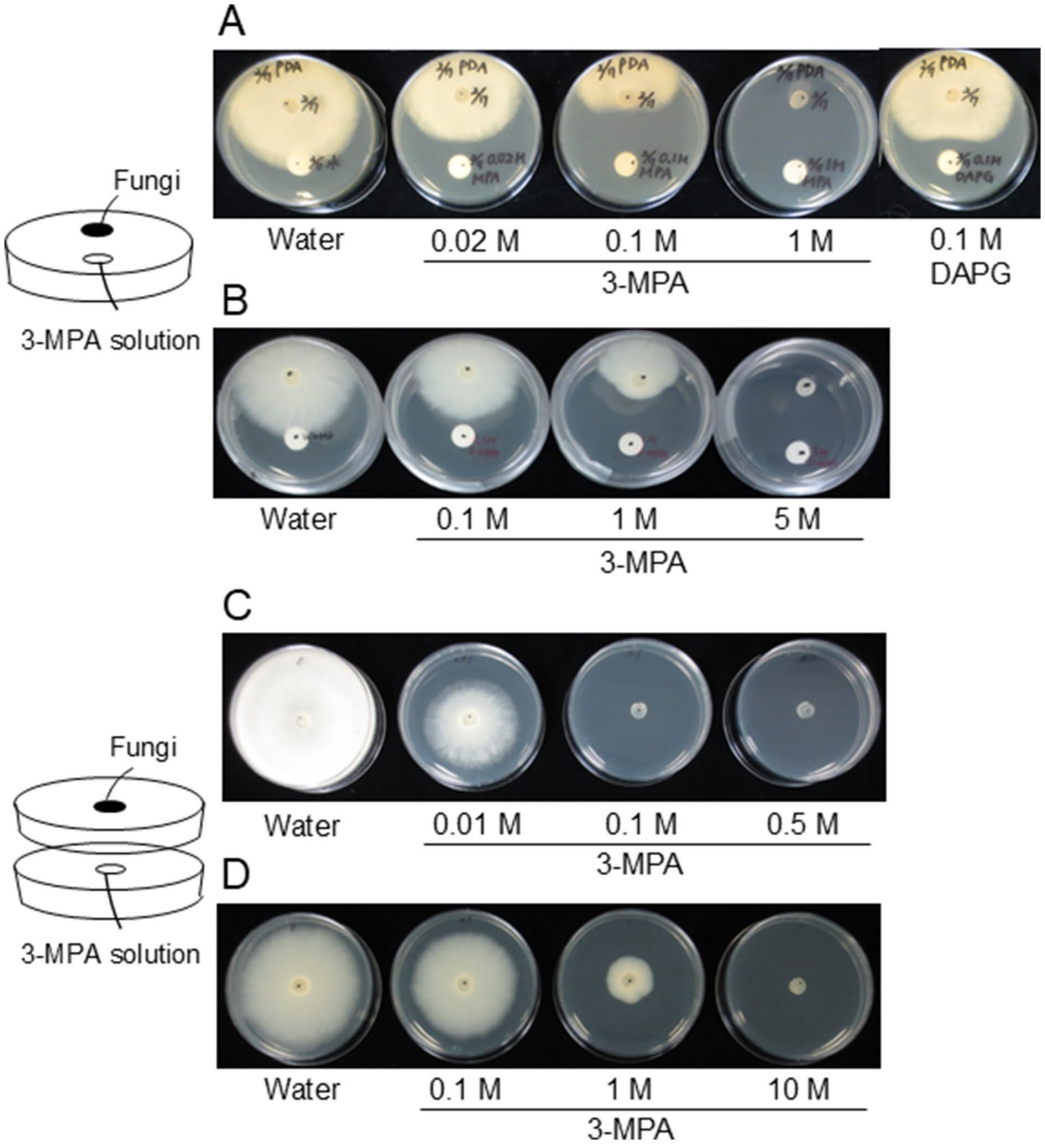
Detection of antibiotic activities of 3-MPA against *P. ultimum* and *F. oxysporum*. The antibiotic activities of 3-MPA against *P. ultimum* **(A)** and *F. oxysporum* **(B)** grown on potato dextrose agar (PDA) plates were evaluated by the distance between filter paper and the filaments of *P. ultimum* or *F. oxysporum*. In each experiment, 30 μL of 3-MPA or 2,4-diacetylphloroglucinol (DAPG) solution of each concentration was spotted onto a round piece of filter paper. The antibiotic activities of volatilized 3-MPA on *P. ultimum* **(C)** and *F. oxysporum* **(D)** were evaluated using the double-plate chamber method. Growth inhibitory rates were assessed by the size of the growth zone of *P. ultimum* or *F. oxysporum*. In each experiment, 100 μL of 3-MPA solution of each concentration was spotted onto a round piece of filter paper. All incubations were performed in triplicate. The results of quantitative assessment of growth inhibitory rates are shown in Supplementary Figure S7.

To investigate the role of 3-MPA in strain CHA0, we initially evaluated the growth inhibitory activities of 3-MPA toward the phytopathogenic oomycete *P. ultimum* and fungus *F. oxysporum*. Mycelial growth was inhibited by 3-MPA in a dose-dependent manner (Figures 2A,B). 3-MPA exhibited stronger antibiotic activity against *P. ultimum* than DAPG. We also investigated whether 3-MPA exhibited antibiotic activity without direct contact with pathogens to monitor the effects of volatilized 3-MPA. Growth inhibitory activity was tested using the double-plate chamber method by incubating pathogens and 3-MPA in the same atmosphere, but physically separated from each other, which is the most widely used method for the *in vitro* assessment of VOC-mediated microbial interactions (Win et al., 2022). As shown in Figures 2C,D, volatilized 3-MPA suppressed mycelial growth in a dose-dependent manner.

### 3.3 3-MPA suppresses *Pythium* damping-off and root rot in cucumber

We then investigated the role of 3-MPA in strain CHA0 in a natural habitat by using a cucumber-*P. ultimum* pathosystem, in which we evaluated the efficacy of disease suppression by counting surviving plants and measuring root and shoot weights. As shown in Table 1, the number of surviving plants and fresh weights were both markedly lower in *P. ultimum*-inoculated plants than in disease-free plants. In contrast, the treatment with 3-MPA significantly alleviated the negative impact of *P. ultimum* infection on cucumber, but did not restore fresh weights to the levels of the mock control. The addition of 3-MPA itself did not affect the number of surviving plants, but reduced fresh weights (Supplementary Table S4). This effect of 3-MPA on fresh weights may have influenced the recovery from *P. ultimum* infection. Plant growth features of cucumber seedlings are shown in Supplementary Figure S5. Again, the level of damping-off and root rot caused by *P. ultimum* was lower when treated with 3-MPA. The addition of 3-MPA itself caused dwarfism in plants, but did not cause yellowing or browning.

### 3.4 3-MPA production is up-regulated in a *3mdo* mutant

**Figure 3:**
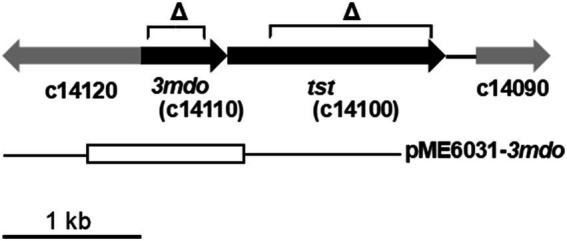
Genomic map of *3mdo* and *tst* genes in *P. protegens* CHA0 and the construction of mutants. *Δ* represents the regions deleted in *3mdo* or *tst*. The open box below indicates the region used for complementation in pME6031 as described under “Materials and Methods”.

Regarding the PA2602 gene encoding 3MDO in the *P. aeruginosa* PAO1 genome (Tchesnokov et al., 2015), we identified its homolog c14110 in the CHA0 genome and hereafter referred to it as *3mdo*. To investigate the role of 3-MPA in CHA0, we generated chromosomal *3mdo* deletion mutants (termed Δ*3mdo*; Figure 3) in a wild-type background of CHA0 for further analyses. We also identified the c14100 gene, which appears to form a transcription unit with the upstream *3mdo* gene (Figure 3). The homolog of the c14100 gene in PAO1 (PA2603) has been annotated as a probable gene encoding thiosulfate sulfurtransferase (TST). We then constructed a *tst* deletion mutant (termed Δ*tst*; Figure 3) in CHA0.

Extracellular 3-MPA levels were assessed in each strain by measuring mBr3MPA after mBr derivatization (Table 3). More 3-MPA accumulated in the *3mdo* mutant than in wild-type CHA0, confirming the predicted function of 3MDO in the dissimilation of 3-MPA. On the other hand, the effect of the *tst* mutation was not significant. Therefore, the phenotype of the *tst* mutant was not subjected to further investigation.

**Table 3:** Comparison of extracellular 3-mercaptopropionic acid (3-MPA) concentrations in *Pseudomonas protegens* CHA0 and its mutant.

Bacterial strain	Genotype	3-MPA (pg/OD_600_)^a^
4 h		
CHA0	Wild type	1457.0 ± 93.6
CHA03mdo	Δ*3mdo*	1670.3 ± 24.3*
CHA0tst	Δ*tst*	1716.3 ± 127.6
8 h		
CHA0	Wild type	1750.4 ± 124.5
CHA03mdo	Δ*3mdo*	2767.5 ± 291.6*
CHA0tst	Δ*tst*	1968.3 ± 127.6

^a^Data are presented as the averages of three replicates ± standard deviations. Samples were collected 4 or 8 h after the start of the incubation. OD_600_ = optical density at 600 nm; * indicates values that were significantly different from those in CHA0 at *p* < 0.05 (*t*-test).

We constructed a plasmid carrying *3mdo* with its promoter region and introduced it into the *3mdo* mutant for complementation. The amount of 3-MPA in the resultant strain decreased, confirming the complemented function of *3mdo* (Table 4). We also measured 3-MPA in the *gacA* mutant to establish whether 3-MPA accumulated in a *gacA*-dependent manner. The level of 3-MPA that accumulated was similar in the *gacA* mutant and wild-type CHA0. These results suggest that the Gac/Rsm system did not regulate the production of 3-MPA under the conditions tested.

**Table 4:** Comparison of extracellular 3-mercaptopropionic acid (3-MPA) concentrations in *Pseudomonas protegens* CHA0 and its mutant.

Bacterial strain	Genotype	3-MPA (pg/OD_600_)^a^
CHA0	Wild type	979.5 ± 162.3
CHA03mdo	Δ*3mdo*	1843.5 ± 736.8*
CHA03mdoC	Δ*3mdo* + *3mdo*	656.4 ± 149.3**^,^ ***
CHA0gacA	Δ*gacA*	1029.3 ± 20.1

^a^Data are presented as the averages of four replicates ± standard deviations. Samples were collected 8 h after the start of the incubation. OD_600_ = optical density at 600 nm; * and ** indicate values that were significantly different from those in CHA0 at *p* < 0.1 and 0.05, respectively (*t*-test); *** indicates values that were significantly different from those in CHA03mdo at *p* < 0.05 (*t-*test).

### 3.5 The *3mdo* mutant exhibits increased antibiotic activity

**Figure 4:**
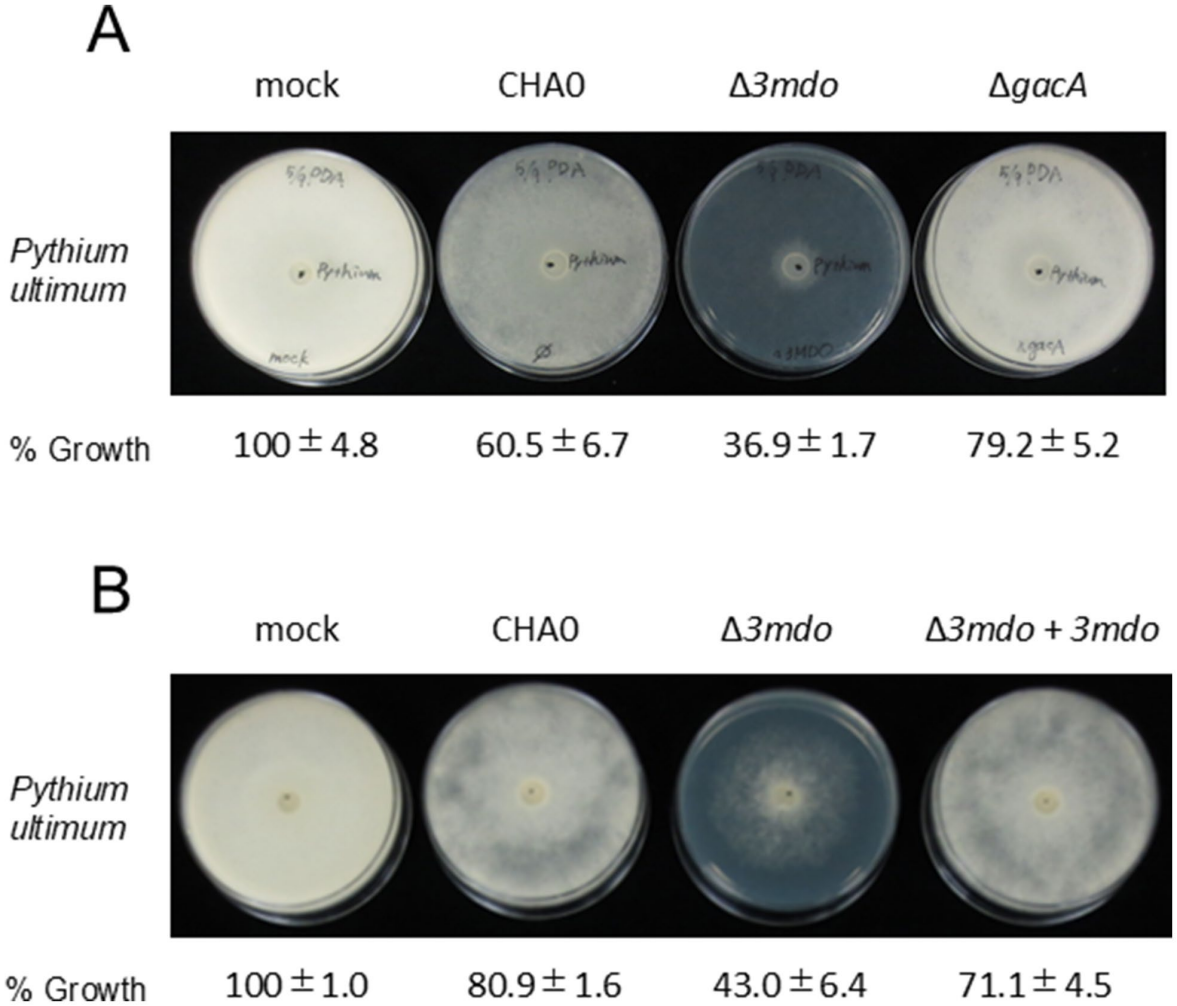
Effects of *3MDO* and *gacA* mutations on antibiotic activity against *P. ultimum*. The GCM plate with a bacterial strain and PDA plate inoculated with *P. ultimum* were placed opposite to each other and sealed with plastic tape. The antibiotic activities of *P. protegens* CHA0 (wild type), the *3MDO* mutant (Δ*3MDO*), and the *gacA* mutant (Δ*gacA*) were compared in panel **A**. The antibiotic activities of *P. protegens* CHA0 (wild type), the *3MDO* mutant (Δ*3MDO*), and *3MDO* complementation strain (Δ*3MDO* + *3MDO*) were compared in panel **B**. Growth inhibitory rates were evaluated by the size and thickness of the growth zone of *P. ultimum*. All incubations were performed in triplicate. The growth levels of *P. ultimum* were quantified using ImageJ software. Data are shown as the averages of 3 replicates ± standard deviation.

We investigated whether the production of 3-MPA affected the antibiotic activity of strain CHA0 by utilizing the *3mdo* mutant and its complement (Figures 4A,B). Growth inhibitory activity against *P. ultimum* was elevated in the *3mdo* mutant, but was decreased in the complemented strain, confirming the complemented function of *3mdo*. These results indicate that the production of 3-MPA affected the antibiotic activity of CHA0. No significant difference was observed in bacterial growth between wild-type CHA0 and the *3mdo* mutant (Supplementary Figure S6). The *gacA* mutant exhibited intermediate growth inhibitory activity between the mock control and wild-type CHA0, suggesting that the effects of antibiotic VOCs, such as HCN, the production of which is regulated by GacA (Blumer et al., 1999), were attenuated (Figure 4A). Consistent with previous findings (van den Broek et al., 2005; Seaton et al., 2013), the mutation in *gacA* enhanced bacterial growth (Supplementary Figure S6).

### 3.6 The *3mdo* mutant exhibits enhanced biocontrol efficacy

To investigate the effects of the *3mdo* mutation in a natural habitat, we used a cucumber-*P. ultimum* pathosystem, in which we evaluated plant protection efficacy by counting surviving plants and measuring root and shoot weights. In terms of biocontrol efficacy, the *3mdo* mutant was more effective than wild-type CHA0 at increasing the number of surviving plants and root fresh weights (with 95% confidence), whereas shoot fresh weights were not significantly different at this confidence level. The wild type and *3mdo* mutant of strain CHA0 did not affect the growth of cucumber in the absence of *P. ultimum* (Table 2).

## 4 Discussion

In the *3mdo* mutant, the level of 3-MPA that accumulated was elevated without affecting bacterial growth, showing enhanced antibiotic activity and plant protection efficacy. Therefore, 3-MPA is an important biocontrol factor of *P. protegens* CHA0. Unlike other antibiotic metabolites, the production of 3-MPA is not under the control of the Gac/Rsm signal transduction pathway. This may be advantageous for designing biocontrol agents because previous studies demonstrated that the overexpression of Gac/Rsm resulted in no improvements or even reductions in the efficacy of plant protection in a natural habitat (Zuber et al., 2003; Takeuchi et al., 2009; Takeuchi et al., 2014). Since the production of 3-MPA increased at both the optimal growth phase and production phase (4 h and 8 h after the inoculation, respectively), the regulatory mechanism of 3-MPA production may not be canonical as typical Gac/Rsm-controlled antibiotics, such as DAPG produced in the production phase.

Limited information is available on the metabolic pathways and enzymes involved in 3-MPA production. 3MDO is the only enzyme that has been reported to play a role in the dissimilation of 3-MPA (Tchesnokov et al., 2015). Regarding the biosynthesis of 3-MPA, the biosynthetic enzyme(s) has yet to be reported; however, a model has been proposed. Some microbes have been suggested to produce 3-MPA as a metabolite of dimethylsulfoniopropionate, methionine, and homocysteine in marine sediments (Kiene and Taylor, 1988); therefore, these candidate 3-MPA precursors may contribute to the identification of genes encoding the enzymes required for the biosynthesis of 3-MPA using a transcriptomic analysis of genes responding to precursors.

3-MPA concentrations in the rhizosphere in natural soil are an important factor that needs to be considered from a biocontrol point of view. 3-MPA is the most abundant thiol in freshwater and marine environments and accumulates as a metabolite of marine bacteria (Visscher and Taylor, 1994; Hu et al., 2006); however, its concentrations in the rhizosphere remain unclear. Its chemical characteristic as a volatile compound may make it difficult to detect in natural soil. The present results on 3-MPA detection in culture supernatants suggest that the concentration of 3-MPA in *P. protegens* CHA0 was in a low ng/OD_600_/mL range, corresponding to a low nM range. Although precise comparisons were not possible because of differences in both the culture conditions and the detection procedures tested, DAPG, MAPG, and pyoluteorin concentrations were previously reported to be in the low μM range (Schnider-Keel et al., 2000), suggesting that 3-MPA was not as abundant as other antibiotic metabolites in the natural habitat. Considering that 3-MPA exhibited stronger antibiotic activity against *P. ultimum* than DAPG (Figure 2A), *P. protegens* CHA0 could suppress plant disease with 3-MPA at the lower level of production in the rhizosphere.

It is important to note that 3-MPA acts as an inhibitor of metallo-*β*-lactamase (MBL), an enzyme involved in antibiotic resistance in bacteria (Goto et al., 1997; Siemann et al., 2003). Furthermore, although it is a well-known natural organic compound, the function of 3-MPA as a bacterial metabolite has yet to be clarified. Previous studies on *M. jannaschii* and *V. paradoxus* revealed the production of 3-MPA as their actual metabolite (Allen and White, 2016; Bruland et al., 2009). In pseudomonads, some strains have been shown to utilize 3-MPA; however, it remains unclear whether they produce 3-MPA. Therefore, this is the first study to describe not only the detection of 3-MPA in a culture of pseudomonads, but also the functions of 3-MPA as an antibiotic metabolite and biocontrol factor.

The identification of genes involved in the 3-MPA biosynthetic pathway and regulatory system warrants further study. From a pharmacological perspective, 3-MPA has been reported to inhibit glutamate decarboxylase in the rat brain and bacterial GABA transaminase (Lamar, 1970). We previously reported that exogenous Glu exerted a positive effect on the chitinase activity and biocontrol efficacy of *P. protegens* (Takeuchi et al., 2023). Furthermore, we demonstrated that the accumulation of GABA played a positive role in the root colonization ability of *P. protegens* (Takeuchi, 2018). These findings suggest the existence of a regulatory link between 3-MPA and favorable metabolites, such as Glu and GABA, in fine-tuning of the behavior of *P. protegens* in the rhizosphere.

To optimize pseudomonad strains for disease control, the loss of 3MDO function will lead to the practical use of this strain. In the present study, 3-MPA was shown to play an important role in the antibiotic activity and biocontrol efficacy of *P. protegens* CHA0. The potential of 3-MPA itself to function as a fungicide in the rhizosphere is also of interest, although we need to consider the dwarfing effects on plants. Thiol-containing compounds have been shown to exhibit pesticide activity. In the context of environmental impact, 3-MPA has been reported to remove toxic metals from water (Morillo et al., 2015). In consideration of the inhibitory activity of 3-MPA against MBLs, 3-MPA will exhibit broad-spectrum disease suppression capabilities against both fungal and bacterial phytopathogens. This inexpensive, low-molecular-weight volatile compound is promising for the suppression of soil-borne diseases.

## Data Availability

The original contributions presented in the study are included in the article/Supplementary material, further inquiries can be directed to the corresponding authors.
