# Supplementary material for: 3-Mercaptopropionic acid as a potential biocontrol factor contributes to plant protection efficacy of Pseudomonas protegens CHA0
Source: Front Microbiol. 2026 Feb 13;17:1726349. doi: 10.3389/fmicb.2026.1726349 (PMC12947264; doi:10.3389/fmicb.2026.1726349)
Supplement: Supplementary file 1 [file Data_Sheet_1.docx]

Supplementary Material

## Supplementary Figures

##

## Supplementary Figure S1. Monobromobimane derivatization of MPA. Monobromobimane (mBr) reacts with 2-MPA and 3-MPA to produce mBr2MPA and mBr3MPA, respectively.

**Supplementary Figure S2.** Standard curves of mBr2MPA and mBr3MPA. Standard curves of mBr2MPA (A) and mBr3MPA (B) were prepared using a set of serial dilutions (0.025, 0.05, 0.25, 0.5, 2.5, and 5 pg).

**Supplementary Figure S3.** Analysis of MPA derivatives by UPLC-MS/MS. 2-MPA or 3-MPA was reacted with mBr, and each reactant was mixed and subjected to UPLC-MS/MS in a scan mode ranging from *m/z* 50 to 500. (**A**) A total ion chromatogram (TIC) of a mixture containing 1 ng each of mBr2MPA and mBr3MPA. Peaks with retention times of 0.58 and 1.75 min were tris(hydroxymethyl)aminomethane (Tris) used as the buffer and free mBr, respectively. Mass spectra of peaks with retention times of 2.34 (**B**) and 3.17 min (**C**).

**Supplementary Figure S4.** Analysis of supernatants of the bacterial culture by UPLC-MS/MS. The spent culture supernatant of strain CHA0 was incubated with mBr, and the reactants were subjected to UPLC-MS/MS. (A) Total ion chromatograph (TIC). (B) Extracted ion chromatogram at *m/z* 297.

**Supplementary Figure S5.** Plant growth features of cucumber seedlings harvested after 14 days. The corresponding data are shown in Table 1 and Supplementary Table S4.

**Supplementary Figure S6.** The growth of *P. protegens* CHA0 and mutants in liquid GCM. Strain CHA0 and the mutants were grown in Erlenmeyer flasks at 180 rpm at 30℃ after the inoculation (scaling up from an overnight culture to a fresh culture, 100-fold). Measurements were performed in quadruple. Symbols indicate averages, and error bars indicate S.D. values. OD_600_, optical density at 600 nm.

**Supplementary Figure S7.** Quantitative assessment of antibiotic activities of 3-MPA against *P. ultimum* and *F. oxysporum*. (A) The antibiotic activities of spotted 3-MPA against *P. ultimum* and *F. oxysporum*. The sizes of growth inhibition zone were measured from the edge of the filter paper to the end of the clear zone. Data are shown as the averages of 3 replicates ± standard deviation. The corresponding photographs are shown in Figures 2A and 2B. (B) The antibiotic activities of volatilized 3-MPA against *P. ultimum* and *F. oxysporum*. The sizes of growth inhibition zone were measured from the edge of the plate to the end of the clear zone. The average of the four sides was calculated in each plate, then the averages of 3 replicates ± standard deviation are shown. The corresponding photographs are shown in Figures 2C and 2D.

## Supplementary Tables

**Supplementary Table S1**. Bacterial strains and plasmids used in the present study.

| Strain or plasmid | Description | Source or reference |
| --- | --- | --- |
| Strains  *Escherichia coli*  DH5α, HB101  *Pseudomonas protegens*  CHA0  CHA0gacA  CHA03mdo  CHA0tst  CHA03mdoC  Plasmids  pCR-Blunt II-TOPO  pME497  pME3087  pME3087gacA  pME30873mdo  pME3087tst  pME6031  pME60313mdo | Laboratory strains  Wild type  Δ*gacA*  Δ*3mdo*  Δ*tst*  Δ*3mdo* strain harboring pME60313mdo  Cloning vector; Km^r^  Mobilizing plasmid; Ap^r^  Suicide vector, ColE1 replicon, Mob; Tc^r^  pME3087 containing the *Eco*RI/*Hin*dIII 1.4-kb *gacA* region with a 0.6-kb deletion in the *gacA* gene; Tc^r^  pME3087 containing a *Kpn*I/*Hin*dIII 1.4-kb *3mdo* region with a 0.4-kb deletion in the *3mdo* gene; Tc^r^  pME3087 containing a *Bam*HI/*Hin*dIII 1.5-kb *tst* region with a 1.2-kb deletion in the *tst* gene; Tc^r^  pACYC177-pVS1 shuttle vector; Tc^r^  pME6031 containing the *3mdo* gene | Sambrook and Russell, 2001  Stutz et al*.,* 1986  The present study  The present study  The present study  The present study  Invitrogen  Voisard et al., 1988  Voisard et al., 1994  The present study  The present study  The present study  Heeb et al., 2000  The present study |

REFERENCES

Heeb, S., Itoh, Y., Nishijyo, T., Schnider, U., Keel, C., Wade, J., et al. (2000). Small, stable shuttle vectors based on the minimal pVS1 replicon for use in gram-negative, plant-associated bacteria. *Mol. Plant Microbe Interact.* 13, 232–237. doi: 10.1094/MPMI.2000.13.2.232

Sambrook, J., and Russell, D. W. (2001). Molecular Cloning: A Laboratory Manual, 3rd Ed. Cold Spring Harbor Laboratory Press, Cold Spring Harbor, NY, U.S.A.

Stutz, E. W., D´efago, G., and Kern, H. (1986). Naturally occurring fluorescent pseudomonads involved in suppression of black root rot of tobacco. *Phytopathology* 76, 181–185. doi: 10.1094/Phyto-76-181

Voisard, C., Bull, C. T., Keel, C., Laville, J., Maurhofer, M., Schnider, U., et al. (1994). Biocontrol of root diseases by *Pseudomonas fluorescens* CHA0: current concepts and experimental approaches. In: Molecular Ecology of Rhizosphere Microorganisms (O'Gara, F., Dowling, D.N., and Boesten, B., eds) pp. 67–89, VCH Weinheim, Germany. doi: 10.1002/9783527615810.ch6

Voisard, C., Rella, M., and Haas, D. (1988). Conjugative transfer of plasmid RP1 to soil isolates of *Pseudomonas fluorescens* is facilitated by certain large RP1 deletions. *FEMS Microbiol. Lett*. 55, 9–13. doi: 10.1111/j.1574-6968.1988.tb02790.x

**Supplementary Table S2.** Oligonucleotides used in the present study.

| Oligonucleotide | Description | Source or reference |
| --- | --- | --- |
| 3mdoUF | 5'-ACGTGGTACCTGGTAACAGTCGGCCAGTTT-3',  underlining indicates the artificial *Kpn*I site | The present study |
| 3mdoUR | 5'-GAAGTTGGCTGATTCGATGG-3', anneals to the 5' region of 3mdoDF | The present study |
| 3mdoDF | 5’-CCATCGAATCAGCCAACTTC ATCCATCAGGTGAGCAATGC-3' | The present study |
| 3mdoDR | 5'-ACGTAAGCTTGCTCATGGTCTGGTATTCG-3',  underlining indicates the artificial *Hin*dIII site | The present study |
| TSTUF | 5'-ACGTGGATCCGCAGCGCTACCAGCAGTT-3',  underlining indicates the artificial *Bam*HI site | The present study |
| TSTUR | 5'-GTTGACGTCCTTGAACAGCT-3', anneals to the 5' region of TSTDF | The present study |
| TSTDF | 5'-AGCTGTTCAAGGACGTCAAC  AAAGAAGCCATGCAGGGCTA-3' | The present study |
| TSTDR | 5'-ACGTAAGCTTCGAAGCGGTGCAGCATA-3',  underlining indicates the artificial *Hin*dIII site | The present study |
| GacAUF | 5'-ACGTGAATTCACGAGCAAATTGTTGAA-3',  underlining indicates the artificial *Eco*RI site | The present study |
| GacAUR | 5'-ATGGTCATCGACTACTAGCA-3', anneals to the 5' region of GacADF | The present study |
| GacADF | 5'-TGCTAGTAGTCGATGACCAT  AGCCTCTGAAATGACCGAAC-3' | The present study |
| GacADR | 5'-ACGTAAGCTTCCATGCTCTGCTGGTCCT-3',  underlining indicates the artificial *Hin*dIII site | The present study |
| 3mdoCompF | 5'-ACGTAAGCTTCATTGGCCACCAGTTCATT-3',  underlining indicates the artificial *Hin*dIII site | The present study |
| 3mdoCompR | 5'-ACGTGAATTCTTCACGCACATCGATCAGT-3',  underlining indicates the artificial *Eco*RI site | The present study |

**Supplementary Table S3**. Detection of 2-mercaptopropionic acid (2-MPA) and 3-mercaptopropionic acid (3-MPA) from the supernatant of *Pseudomonas protegens* CHA0.

| **2-MPA (pg/OD_600_)^z^** | **3-MPA(pg/OD_600_)^z^** |
| --- | --- |
| 56.7 ± 43.3 | 1664.4 ± 99.2 |

^z^ Data are presented as averages of three replicates ± standard deviations. OD_600_ = optical density at 600 nm.

**Supplementary Table S4**. Evaluation of surviving plants and fresh weights of cucumber with the 3-MPA treatment.

| 3-MPA added* | Surviving plants per pot (%)** | Shoot fresh weight  per pot (g)*** | |  | Root fresh weight  per pot (g)*** | |
| --- | --- | --- | --- | --- | --- | --- |
|  |  | 1^st^ Exp. | 2^nd^ Exp. |  | 1^st^ Exp. | 2^nd^ Exp. |
| None | 100 a | 0.42 a | 0.46 a |  | 0.09 a | 0.12 a |
| 2 mM | 100 a | 0.30 b | 0.33 b |  | 0.08 a | 0.12 a |
| 5 mM | 100 a | 0.25 c | 0.24 c |  | 0.05 b | 0.05 b |

* One milliliter of 3-MPA of 200 or 500 mM was added to 300 ml of vermiculite absorbed in 100 ml of water to make the final concentration of 2 mM or 5 mM 3-MPA in water (50 ml of soil per pot) before planting. Plants were harvested after 14 days. The corresponding photographs of growth features of cucumber seedlings are shown in Supplementary Figure S5.

** Data represent the means of two individual repetitions of the same experimental set-up, with 6 replicates (pots contain three cucumber plants) per treatment in each experiment and 12 replicates (pots) in total. Means within the same column followed by different letters (a to c) are significantly different (*P* <0.05) according to Tukey’s honestly significant difference (HSD) test.

*** Data represent the means of 6 replicates (pots contain three cucumber plants) per treatment in each experiment. Means within the same column followed by different letters (a to c) are significantly different (*P* <0.05) according to Tukey’s honestly significant difference (HSD) test.
